# Implementation of the laboratory quality management system (ISO 15189): Experience from Bugando Medical Centre Clinical Laboratory – Mwanza, Tanzania

**DOI:** 10.4102/ajlm.v7i1.657

**Published:** 2018-07-31

**Authors:** Medard Beyanga, Lisa Gerwing-Adima, Kahima Jackson, Benjamin Majaliwa, Henrico Shimba, Simon Ezekiel, Charles Massambu, Dickson Majige, Michael Mwasegaka, Wilson Mtotela, Patrick Mateta, Christa Kasang

**Affiliations:** 1 Department of Clinical Laboratory Services, Bugando Medical Center, Mwanza, Tanzania; 2 Tanzania Ministry of Health Community Development, Gender, Elderly and Children, Dodoma, Tanzania; 3 US Centers for Disease Control and Prevention, Dar es Salaam, Tanzania; 4 Clinical and Laboratory Standards Institute, Wayne, New Jersey, United States; 5 Medical Mission Institute, Wuerzburg, Bavaria, Germany

## Abstract

**Background:**

Use of laboratory evidence-based patient health care in Tanzania remains a complex problem, as with many other countries in sub-Saharan Africa. As at 2010, 39 African countries, including Tanzania, had no clinical laboratories that met the minimum requirements for international laboratory standards (International Organization for Standardization [ISO] 15189).

**Objective:**

The aim of this article is to share experience from Bugando Medical Centre laboratory’s milestones in reaching ISO 15189 accreditation.

**Methods:**

Mentors to address the laboratory management and technical requirements performed a gap analysis using the Southern African Development Community Accreditation system checklist. Several non-conformances were detected. System and technical procedures were developed, approved and communicated. Quality indicators were established to measure laboratory improvement and to identify issues which require immediate and preventive actions.

**Results:**

The departments’ external quality assessment performance increased after ISO 15189 implementation (e.g. Parasitology from 45% to 100%, Molecular Biology from no records to 100%, Biochemistry 50% to 95%, Tuberculosis Microscopy 60% to 100%, and Microbiology from 48.1% to 100%). There was a reduction in complaints, from eight to two per week. Rejected samples were reduced from 7.2% to 1.2%. Turn-around time was not recorded before implementation but reached 92% (1644/1786) of the defined targets, and the proportion of contamination in blood cultures decreased from 16% to 4%.

**Conclusion:**

Our experience suggests that the implementation of a quality management system is possible in resource-limited countries like Tanzania. Mentorship is necessary and should be done by professional laboratory mentors trained in quality management systems. Financial resources and motivated staff are key to achieving ISO 15189 accreditation.

## Background

The use of laboratory evidence-based patient healthcare in Tanzania remains a complex problem, as in many other countries in sub-Saharan Africa.^1^ Clinicians often believe that laboratory tests are additional health costs, because diagnosis and treatment are often done using empirical clinical judgement. Laboratory results are perceived to be unreliable, especially when they are found to be discordant with clinical indications.^2^

It has been emphasised that healthcare professionals should make clinical decisions based on the best available evidence.^3^ Access to unreliable diagnostic services and misdiagnosis causes confusion during patient management, and can result in unnecessary expenditure and in some cases death.^4^ There are recent global calls to provide more resources for the diagnosis, treatment and prevention of infectious diseases affecting the African population. In contrast, clinicians, financial controllers and public policy makers in resource-limited settings may be unaware of the importance of a laboratory-proven diagnosis for appropriate disease management.^4^

There is sufficient evidence that laboratory services are essential for guiding patient care. However, their central role has been neglected in resource-limited countries for decades.^5^ Laboratory results should guide about 70% of clinical decisions and promote excellence in providing the best patient care. Provision of accurate and reliable laboratory test results is only possible if the laboratory meets a minimum standard that provides credibility of the reported test results.^6^

Until 2010 there were 380 laboratories which met international standards for quality in Africa.^7^ Tanzania, along with 38 of the 49 African countries assessed, had no clinical laboratories that met the minimum requirements for international quality standards to ensure reliability of reported patient results and competence of staff.^7^

The Maputo Declaration of 2008 pointed out that strengthening of laboratory systems requires collaboration between laboratories, governments and supporting partners.^8^ This collaboration can only be met by creating and implementing individual country plans and strategies which utilise the laboratory for diagnosis of diseases.^9^

Before the Maputo Declaration, the Ministry of Health Community Development, Gender, Elderly and Children, in collaboration with the Clinical & Laboratory Standards Institute (CLSI), and with support from the President’s Emergency Plan for AIDS Relief, had been working together to implement quality management systems (QMS) in five zonal referral hospital laboratories. Bugando Medical Centre Laboratory was among the five referral hospital laboratories.^10,11^

The aim of this article is to share experience from the Bugando Medical Centre laboratory’s journey to International Organization for Standardization (ISO) 15189 accreditation. The experience will inform most laboratories currently implementing QMS to refamiliarise themselves with the actual steps required before inviting assessors to assess laboratories and give recommendations for ISO accreditation.

## Methodology

### Ethical considerations

We used data from routine laboratory QMS operations. No patient information was used; thus there was no ethical review required for this article.

### Gap analysis

The laboratory gap analysis was done for both management and technical areas of the laboratory to establish baseline data. The approach was to evaluate current laboratory practices and compare it with laboratory standards as stipulated by ISO. The gap analysis revealed numerous non-conformances after comparing actual performance with the standard. The gaps identified included lack of knowledge about the laboratory standard, insufficient technical skills among staff and the use of unverified technical methods. Other gaps were: absence of established policies and technical procedures, samples tested by staff who were deemed incompetent, and lack of staff training and supervision to carry out a test (see Box 1).

Box 1 List of gaps that were identified during the gap analysis.

- Quality Officer had not officially been appointed and had no job description.
- Quality documents had not been fully communicated to all laboratory staff.
- Purchased equipment and consumables supplies were checked for quality by examining Quality Control samples but results were not verified.
- There were no records of evaluated suppliers of critical reagents, supplies and services.
- Records of complaints from clinicians, patients and other parties, together with action plans developed, were available, but there was no evidence that they had been shared with the entire laboratory staff.
- No action plan was developed when there was a need to perform preventive action in order to reduce the likelihood of the occurrences.
- Action plan developed from management review meeting did not include goals and objectives.
- No evidence that findings and actions that arose from management review had been communicated to laboratory staff.
- Deputies for all key functions were not officially appointed.
- There was no evidence that staff had been trained and thereafter checked for competency.
- There was no procedure for shutdown of the computer in event of software and / or hardware failure.
- There was no policy which defined levels of access when using laboratory computers.
- There was no procedure to evaluate requests and samples.
- There was no specific report form.
- There was no system in place to ensure patients’ reports are dispatched in time.
- There was no evidence that safety minutes are available in the laboratory.
- Relevant clinical data for tests was not recorded on all request forms.
- Date and time of specimen collection were not recorded on the request forms.
- Sample collection manuals were not available at all primary sample collection sites.
- Turn-around time for laboratory tests were not monitored, since the specimen collection time was not recorded.
- There was no evidence that samples transported to the laboratory had not been exposed to temperatures outside of an acceptable range.
- There was no evidence that samples had been transported safely to the laboratory in compliance with safety requirements.
- During assessments, the sample collection manual was not in place at the reception area; thus, instructions were not followed.
- Not all equipment was uniquely labelled.
- Most laboratory equipment had no manufacturer’s instructions/ manuals.
- There were no performance records to confirm that equipment continued to be suitable for use.
- Laboratory equipment had no preventive maintenance schedules, except for safety cabinets.
- Some equipment had no operating manuals, especially old equipment.
- There were no records that safety checks on instruments were carried out at regular intervals.
- There were no procedures or records showing that equipment was decontaminated prior to service.
- There were no records that all software were validated.
- There were no procedures in place for protecting the integrity of equipment data.
- There were no records showing that computers and automated equipment are maintained so as to ensure the integrity of data obtained.
- Internal quality control procedures were not evident for all tests done in the laboratory.
- Not all EQA reports were monitored, even though there was no evidence that EQA materials were handled as patient samples.
- There was no evidence that inter-laboratory results were documented or monitored.
- Some tests were performed by personnel who had not been assessed for competency.
- Relevant records of education, professional qualification and registration with professional bodies were not present in some staff personnel files.
- A list of approved signatories for each discipline in the laboratory was not in place.

EQA, external quality assurance

### Implementation of the quality management system

Implementation of the QMS started with management: the hospital management appointed the Laboratory Director, Laboratory Manager and Quality Manager, whereafter the Laboratory Manager appointed section heads to form the laboratory management team as recommended by ISO. The laboratory organisational structure was set primarily to create lines of authority, reporting and communication, and to avoid overlapping of tasks during the QMS implementation process. The Laboratory Manager and Quality Manager attended several QMS training sessions which were organised by the Ministry of Health Community Development, Gender, Elderly and Children in collaboration with CLSI. These trainings were followed by awareness meetings that involved hospital management to make sure that they understood the processes and to ensure the availability of the resources required to enable the QMS implementation process. Hospital management became supportive after gaining a clear understanding of the advantages that a proper and functional QMS offers to both patients and the hospital itself.

### Laboratory mentorship and document development

A laboratory mentor and an advisor were identified to guide laboratory management during the development of laboratory policies and procedures. The mentor was an employee of CLSI, the organisation that was supporting the system implementation. He was a laboratory professional who had a Master’s degree in business administration, and was trained in QMS mentorship and laboratory audits. The advisor was a biomedical laboratory scientist with a Master’s degree in public health and infectious disease. Available laboratory policies and procedures were formalised; those from outside the laboratory were adapted and contextualised to the laboratory setting to form the laboratory manual. The established policy manual formed the basis of other procedures, including the laboratory’s QMS procedures. The laboratory system procedures (Box 2) were developed to cover all quality system essentials, as directed by ISO,^12^ while documentation of technical procedures provided standardisation of all laboratory tests. Developed documents were approved by the Laboratory Director and communicated to all laboratory staff.

Box 2 Laboratory quality management procedures.

1. Document control.
2. Corrective actions.
3. Identification and control of non-conformances.
4. Selection, purchase and equipment management.
5. Sample management.
6. Internal audit.
7. Resolution of complaints.
8. Review of internal quality controls.
9. Methods verification and validation.
10. Estimating measurement of uncertainty.
11. External services and supplies.
12. Results management.
13. Conducting management review.
14. Post exposure prophylaxis.
15. Confidentiality and conduct undertaking.
16. Staff training and competency assessment.
17. Service agreement.
18. Selecting referral laboratories.
19. Inventory controls.
20. Preventive actions.
21. Records control.
22. Use of computers.
23. Review of external quality assessments.

Apart from implementation of laboratory quality essentials (all processes in the laboratory workflow that provide the building blocks of quality, e.g. document control),^13^ the laboratory prioritised four components, which included staff training and competency, methods validation and verification, practising internal quality control in each testing procedure performed within the laboratory, as well as enrolment in external quality control programme schemes. Laboratory management established quality indicators to measure the laboratory’s improvement and to identify corrective and preventive actions.

### Staff training and competency assessment

The laboratory developed a procedure to guide management in conducting training and assessing staff competencies. During the policies training session, the Quality Officer would first address the clause within ISO, then would make sure it had been properly interpreted among laboratory staff and adequately documented within the laboratory policy manual. System procedures were introduced to explain step-by-step approaches to ensure implementation among laboratory staff. Quality management system forms were designed to provide evidence of implementation of procedures, as documented in the laboratory policy manual.

Training sessions were concluded with ‘quizzes’ to check understanding of the discussed procedures. Staff that scored below 80% on the quizzes given after training were retrained and reassessed for competencies. The responsibility for training on technical procedures was assigned to heads of sections, who provided step-by-step guidance on technical procedures. Before staff were allowed to carry out a test, they were assessed by testing known samples, internal controls and external quality assessment materials for reproducibility of results, as well as being checked for all related activities for the respective procedures before being deemed competent. Staff competence procedures also included examination related to scores of 80% on tests. Heads of sections and the Laboratory Manager declared staff to be competent and any deviation of the expected requirements prompted retraining. The Quality Officer took responsibility for adequate implementation and filing of competence forms in personnel files.

### Method validation and verification

The laboratory developed procedures to guide staff on how to validate or verify technical methods; the procedures clearly clarified the difference between verification and validation. Verification was defined as processes that are carried out to prove that the method can provide intended use only when capability data are available; it referred to standard methods used without modification. Validation^14^ was defined as processes that are carried out to prove that the method is fit for the laboratory intended use when no capability data are available; it referred to non-standard laboratory designed methods, which are subjected to subsequent modification. Therefore for validation, the laboratory has to set criteria to accept or reject the test method.

### Qualitative tests

Samples from previously reported results were used to verify methods. Samples were selected randomly (diseased and non-diseased samples) and assigned, in a blinded study, to two different staff members (verifier 1 and verifier 2) deemed competent to carry out the test under verification. Staff carrying out the test were not aware of the results and were supervised, to avoid sharing of results. They were subjected to a similar testing set-up and provided with the method to conduct samples testing. The results produced were again compared to the expected results, also the sensitivity and specificity of the test methods were calculated. The verification results which met the set criteria or manufacturer claims provided sufficient evidence to prove the suitability of the test methods. The verification results which did not meet the criteria demonstrated unfitness of the method for testing of patients.

### Validation or verification of quantitative tests

Accuracy, precision and linearity were determined for individual test methods. Manufactured quality control material was used during the validation or verification process, and control samples were analysed in duplicate three times a day for five days. Precision was calculated to determine coefficient of variation (%), and accuracy was determined to obtain ranges at 95% confidence. The ranges calculated were compared to that of the manufacturer. Linearity was performed by doing serial dilutions on control samples; the diluted samples were tested using the method under verification process, and the results obtained were analysed to obtain the linear line, which was then compared to that of the manufacturer. The laboratory management agreed that the method was fit for testing patients if an acceptable linear line could be visualised after analysis.

### Internal and external quality assessments

The laboratory, with assistance from funding provided by the President’s Emergency Plan for AIDS Relief, procured internal quality control samples and enrolled in external quality assessment (EQA) schemes. Internal quality controls were performed as documented in the established internal quality control procedure. Laboratory staff were trained to evaluate the results of internal controls before they report a patient’s results. The laboratory received EQA samples from Thistle (South Africa), One World Accuracy (Canada), the National Quality Assessment Scheme (United Kingdom), the National Health Laboratory Quality Assurance & Training Centre (Tanzania) and the United States Centers for Disease Control and Prevention (United States).^15^ Samples were received and processed within the deadline, and results from EQA providers were reviewed to identify opportunities for improvement. EQA scores were set to be 80%; when this score was not achieved, it was documented as a non-conformance event, which required root cause analysis and implementation of an identified corrective action.

### Quality indicators

Quality indicators were defined as any measure of the system whereby data collected in a specified period are analysed to determine the improvement of the established system.^16^ Examples of quality indicators which were established to measure laboratory improvement included: external quality assurance performance; number of customer complaints; sample turn-around time; number of rejected samples; equipment downtime; and blood culture sample contamination rate. The laboratory set targets were: (1) EQA performance above 80%, (2) five customer complaints per week, and (3) 90% of samples received to be tested and reported on within the established turn-around time. Turn-around time was defined as time taken from receiving a sample in the laboratory until dispatch of results to a patient. Neither rejected samples nor blood culture contamination should exceed 3% of the total samples received. Quality indicators were reviewed by section members, chaired by the heads of sections and the Quality Officer, and then in quarterly intervals by the quality assurance committee. Deviations from set targets prompted improvement actions.

## Results

External quality assessment (percentage score) for the Parasitology section increased from 45% before implementation of ISO to 100% after implementation, the Biochemistry section performance increased from 50% to 95%, the Molecular biology section performance increased from no record to 100%, the Tuberculosis Microscopy section increased from 60% to 100%, and the Microbiology section increased from 48.1% to 100% (Table 1). The number of complaints decreased from eight to two complaints per week. Rejected samples decreased from 7.2% (18/250) to 1.2% (3/240). The overall turn-around time performance was not recorded before implementation, but reached 92% (1644/1786) of the defined targets, and contamination of blood culture samples decreased from 16% (25/160) to 4% (5/116).

**TABLE 1:** Quality indicators before and after implementation of ISO 15189, Bugando Medical Centre Clinical Laboratory, Tanzania.

Quality indicators	Before QMS implementation (%)	After QMS implementation (%)
EQA performance		
• Parasitology	45	100
• Biochemistry	50	95
• Tuberculosis	60	100
• Microbiology	48.1	100
Molecular Biology	No data	100
Complaints per weeks	80	20
Rejected samples	7.2 (18/250)	1.2 (3/240)
Turn-around time	No data	92 (1644/1786)
Blood culture contamination	16 (25/160)	4 (5/116)

EQA, external quality assurance; QMS, quality management system

**FIGURE 1:**
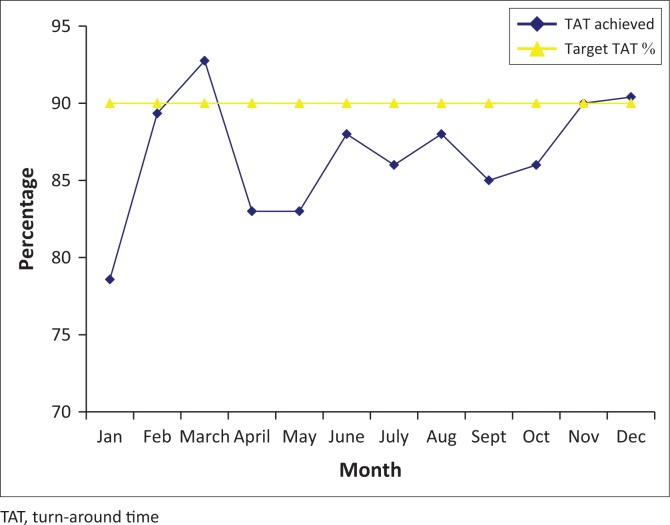
Parasitology turn-around times from January to December 2016. Gap analyses were conducted in January 2012, and implementation of laboratory quality improvement activities started immediately to address the gaps. Accreditation was awarded in March 2014 and indicators have been monitored since.

**FIGURE 2:**
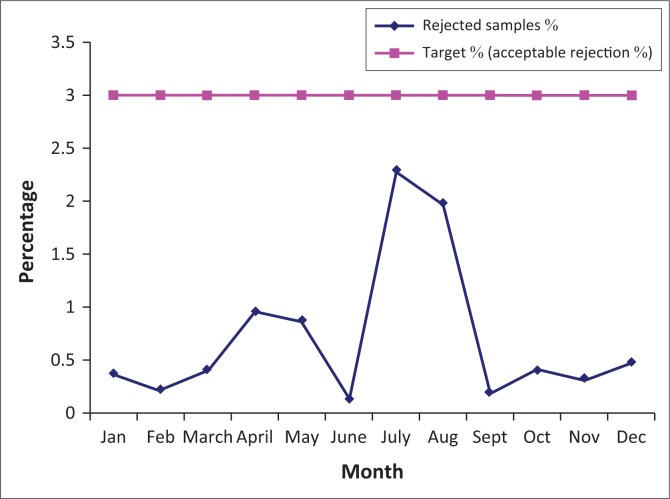
Chemistry sample rejection from January to December 2016.

**FIGURE 3:**
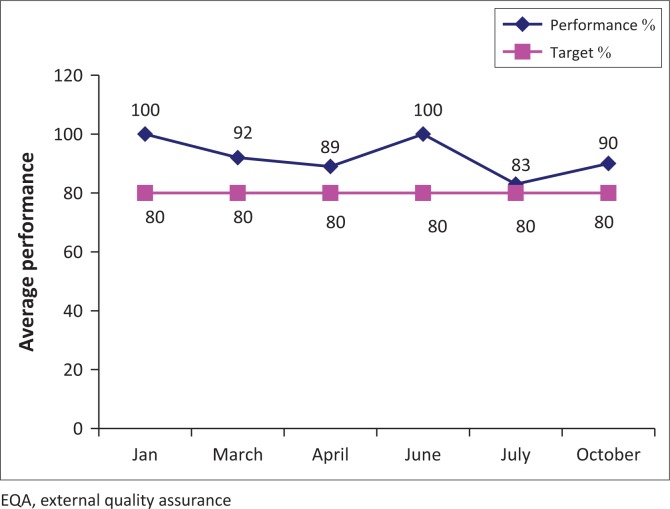
Microbiology external quality assessment January–December 2016.

The gap analysis was conducted on 23 January 2012, the implementation started immediately to address the gaps obtained and accreditation was awarded on 26 March 2014. After QMS implemented laboratory quality improvement activities and monitoring of indicators were possible as displayed in Figures 1–3.

### Accreditation

After 24 months of intensive work, including mentoring activities, the laboratory was ISO 15189 accredited under a Southern African Development Community Accreditation Services/South African National Accreditation System (SADCAS/SANAS)^17^ partnership, with registration number MD002. Tests in five sections were accredited, including Microbiology, Parasitology, Molecular Biology, Biochemistry and Tuberculosis Microscopy. The laboratory witnessed visible advantages of implementing QMS. For example, work done being appreciated by customers, passing EQAs, decreased turn-around times and fewer complaints (Table 1), through monthly monitoring and review of established quality improvement indicators.

## Discussion

Implementation of ISO 15189-based QMS in a routine clinical laboratory at the tertiary hospital, Bugando Medical Centre, was successful in preparing the laboratory for accreditation after 24 months of intense effort. The initial anxiety of staff was counteracted by supportive visits by the Ministry of Health and mentors and constructive advice. The newly-established heads of sections, Quality Officer, Laboratory Manager, Laboratory Director and top hospital management were informed to support the programme. However, the implementation procedures required close follow-up of staff to ensure adherence to the established system, which was not always easy. The laboratory management realised that implementation of QMS also provided regular objective assessment through internal and external audits, ensuring continous improvement in operations. Regular mentorship and a Quality Officer with strong leadership skills transformed the laboratory into a quality-driven organisation. A customer survey, conducted to measure what clinicians were saying about the laboratory services, revealed that clinicians of the hospital were able to trust and make clinical decisions supported by laboratory results.^10^ This was also observed in Botswana, where laboratories with mentorship, support from hospital management and ‘strong lab staff camaraderie’ supported the implementation process.^18^

Similar findings were observed in laboratories in Zimbabwe and Kenya, where mentorship played an important role in the successful implementation of the ISO standard.^19,20^ The laboratory experienced a marked increase of intrinsic motivation during the presence of external CLSI mentors, and the team started to demonstrate a high level of commitment, working beyond official hours without any financial expectations. Following successful accreditation, the Quality Officer observed a sudden decline in commitment of the team, as described in the life cycle of an organisation.^21^ Staff relaxed after their initial hard work, and the Laboratory Manager and Quality Officer were challenged to ensure timely intervention to avoid losing the achieved goals. The laboratory management ensured translation of the achieved goals into daily laboratory operations with its advantages to patient care. There may be a time where a short-term mentor must come in again, as was done in Zimbabwe, when trying to identify an optimal model for mentorship.^22^

Our observations are similar to that reported by a tuberculosis laboratory in Kisumu, Kenya, where culture contamination rate decreased from 15.4% to 5.3% compared to the reduction from 16% to 4% observed in our laboratory before and after implementation of the QMS. The only difference is that the laboratory in Kenya was implementing the standard in a tuberculosis laboratory and was on the implementation process, thus was not accredited as yet.^23,24^ Similar results were achieved by the Strengthening Laboratory Management Towards Accreditation programme in 47 African countries, where the programme is implementing its activities. It was observed in five years of operation that the Strengthening Laboratory Management Towards Accreditation approach formed a unique capacity to assist laboratories to make progress in improving quality of services as a way to achieve accreditation. However, our implementation of similar activities took 24 months of intensive work.^17,25^

### Conclusions

Our experience suggests that implementation of a QMS is possible in resource-limited countries like Tanzania, if adequately supported with human and financial resources. To achieve this level of standard requires trained and well-motivated laboratory staff to implement the system. Mentorship is necessary and should be done by laboratory professionals trained in QMS, who have implemented the system. Financial resources and motivated staff are key to achieving ISO accreditation.
